# Effects of aqueous leaf extract of *Asystasia gangetica* on the blood pressure and heart rate in male spontaneously hypertensive Wistar rats

**DOI:** 10.1186/1472-6882-13-283

**Published:** 2013-10-26

**Authors:** Pierre Mugabo, Ismaila A Raji

**Affiliations:** 1 Discipline of Pharmacology, School of Pharmacy, University of the Western Cape, Private Bag X17, Bellville 7535, South Africa; 2 Division of Basic Medical Sciences, Faculty of Medicine, National University of Science and Technology, PO Box AC 939, Ascot, Bulawayo, Zimbabwe

**Keywords:** *Asystasia gangetica*, Blood pressure, Heart rate, Spontaneously hypertensive rats, Renin angiotensin aldosterone system

## Abstract

**Background:**

*Asystasia gangentica* (*A. gangetica*) belongs to the family Acanthaceae. It is used to treat hypertension, rheumatism, asthma, diabetes mellitus, and as an anthelmintic in South Africa, India, Cameroun, Nigeria, and Kenya respectively. It has also been reported to inhibit the angiotensin I converting enzyme (ACE) *in-vitro*. Therefore, the aim of this study is to investigate the *in-vivo* effect of aqueous leaf extract (ALE) of *A. gangetica* on the blood pressure (BP) and heart rate (HR) in anaesthetized male spontaneously hypertensive rats (SHR); and to elucidate possible mechanism(s) by which it acts.

**Methods:**

The ALE of *A. gangetica* (10–400 mg/kg), angiotensin I human acetate salt hydrate (ANG I, 3.1–100 μg/kg) and angiotensin II human (ANG II, 3.1–50 μg/kg) were administered intravenously. The BP and HR were measured via a pressure transducer connecting the femoral artery to a Powerlab and a computer for recording.

**Results:**

*A. gangetica* significantly (p<0.05), and dose-dependently reduced the systolic, diastolic, and mean arterial BP. The significant (p<0.05) reductions in HR were not dose-dependent. Both ANG I and ANG II increased the BP dose-dependently. Co-infusion of *A. gangetica* (200 mg/kg) with either ANG I or ANG II significantly (p<0.05) suppressed the hypertensive effect of both ANG I and ANG II respectively, and was associated with reductions in HR.

**Conclusions:**

*A. gangetica* ALE reduced BP and HR in the SHR. The reduction in BP may be a result of actions of the ALE on the ACE, the ANG II receptors and the heart rate.

## Background

A great number of plants have been used throughout the world from time immemorial for the prevention and cure of sickness [1]. About eighty percent of the active compounds of drugs used for the treatment of cardiovascular conditions are extracted from medicinal plants, e.g. foxglove (*digitalis lanata*), *Allium sativum* (garlic), camphor and *Adonis vernalis*[1-3].

Drugs used for the treatment of hypertension include angiotensin converting enzyme inhibitors (ACEIs), alpha_1_ adrenoceptor blockers, calcium channel blockers, angiotensin II (ANG II) receptor antagonists, diuretics and beta adrenoceptor blockers [4-7]. Most of these drugs are expensive, and in many cases, none of them can control hypertension on its own. Consequently, combination therapy is often required [5,6]. However, combination therapy (polytherapy) has its drawbacks, such as increased risk of drug interactions, side effects and poor compliance [8-10]. These necessitate continuous search for novel agents which are more effective, cheaper, come with less side-effects, and preferably used alone.

*Asystasia gangentica* (*A. gangetica*) belongs to the family Acanthaceae [11,12]. There are eight species native to Southern Africa, and notably, the Kwazulu Natal province of South Africa [12]. *A. gangetica* is used to treat asthma in Nigeria [13], as an antihelmintic in Kenya [14], and as stomachic, astringent, and diaphoretic in India [15]. It has also been suggested to exhibit antihypertensive effects through inhibition of ACE *in-vitro*[12]. Therefore, the aim of this study was to evaluate the effect of ALE of *A. gangetica* on the blood pressure (BP) and heart rate (HR) in spontaneously hypertensive rats (SHR), and also investigate if inhibition of the ACE or the ANG II receptor mediates its effect *in-vivo*.

## Methods

### Study design

The study was designed as an *in- vivo* experimental model assessing the effects of *A. gangetica* on BP and HR in male SHR.

### Collection and preparation of plant material

*A. gangetica* plants were obtained from Newplant nursery in George, Western Cape, South Africa (SA), in March 2009. A sample of the plant was deposited at the University of the Western Cape (UWC) herbarium for identification and authentication by the taxonomist. It was registered under the voucher number 3469. Fresh leaves (1314.27 g) were picked from plants and washed twice with distilled water. Leaves were permitted to dry completely in a room where the temperature was maintained at 23°C for a period of 14 days. Dried leaves (967 g) were then pulverized to a fine powder (738 g) using a Hammer mill and stored in airtight glass containers.

### Aqueous extraction of plant material

A Soxhlet apparatus was used to extract the active compounds from 738 g powder over 48 hours. A Soxhlet thimble was constructed with glass fibre. Fine powder of the *A. gangetica* was wetted with distilled water, and placed between two layers of glass fibre (used as a barrier to prevent powder from passing into the extract). Distilled water (400 ml) was placed in a round bottom flask and connected together along with the reflux condenser. A heating element was used to supply sufficient heat to boil the distilled water. After extraction, the remnant of the crude plant material was discarded. The aqueous extract was then placed in a deep freezer for a period of 72 hours and transferred to a freeze drier for a period of 72–96 hours to produce a dry powder ready for reconstitution and administration. A powder weighing 179 g was obtained.

### Materials and equipments used in the extraction process of the plant

The materials and equipments used for plant extraction are of standard analytical grade, and include an oven, scissors, weighing balance, glass fibre, Soxhlet extractor, rotovapour (Bachi Rotavapor R200, Switzerland), freeze-drier (Virtis FreezeMobile 12SL, SA), 0.45 μm filter paper (Schleicher & Schuell MicroScience, SA) and a −85°C freezer (Snijder Scientific, SA).

### Animals

Healthy male SHR weighing 250–400 g and aged less than 4 months old were used. The SHR were obtained from the Animal Unit of the University of Cape Town, Cape Town (CPT), SA; housed in the animal room of the School of Pharmacy, UWC; and allowed feed and water *ad-libitum*. The animal room temperature was kept at 24°C, with a 12:12-h light–dark cycle.

### Materials and equipments used in the in- vivo experiments

BP transducer (AD Instruments, CPT, SA), BP amplifier (ML117 AD Instruments, CPT, SA), PowerLab 4/20 T (AD Instruments, Lasec, CPT, SA), computer desktop unit, temperature probe (AD Instruments, Lasec, CPT, SA), Chart 5.0 for Windows software (AD Instruments, Lasec, CPT, SA), Ascor AP 22 syringe pump (United Scientific, CPT, SA), heated rat operating table (BioScience, CPT, SA), overhead lamp, bulldog clamps, 0.5 mm (arterial and venous) cannula, three-way tap, syringe, threads, oxygen mask, surgical scissors and cotton wool.

### Chemicals and drugs used in the in-vivo experiments

Sodium chloride (Adcock Ingram, SA), dimethyl sulfoxide (DMSO) (Merck Chemicals, SA), 0.1% heparin sodium (1000 iu/ml, Intramed, SA), 6% sodium pentobarbitone (Kyron Laboratories, Johannesburg, SA). 0.9% Sodium chloride [16] and/or DMSO [17], were respectively used as vehicle to obtain homogenous dilution of *A. gangetica*, heparin, and the unknown drug. All drugs, except where otherwise indicated, were purchased from Sigma Aldrich, SA. ANG I and ANG II which have potential antagonistic effect to that of *A. gangetica*, were used as negative controls of *A. gangetica*[6,7,18]. Fresh solutions were made at the beginning of each experiment. Chemicals, after having been diluted were kept in ice during the course of the experiment to keep them stable.

### Animal preparation and recording of experimental parameters assessed

After the induction of anesthesia with sodium pentobarbitone (40 mg/kg) intraperitoneally, the rat was placed in a supine position on a heated operating table to maintain the temperature of the body temperature at ±37°C. A temperature probe was inserted into the rectum and used to measure the core temperature via the amplifier connected to the PowerLab® and monitored with a computer throughout the experiment. Tracheotomy was performed and a tube placed in the trachea for easy breathing. Rats were supplied with oxygen through a facial-oxygen mask to keep the air environment surrounding the tracheal cannula rich in oxygen. The external jugular vein and the femoral artery were cannulated with catheters containing normal saline/heparin solution in the ratio of 9 ml to 1 ml, for the intravenous infusion of known drugs and/or the ALE of *A. gangetica*. In experiments where *A. gangetica* was co-administered with a control drug, both external jugular veins were cannulated for simultaneous intravenous administration. One of the femoral arteries was cannulated for measurement of arterial BP [19]. BP was measured as systolic pressure (SBP), diastolic pressure (DBP), and mean arterial pressure (MAP). Both BP and HR were monitored continuously on a computer running the Chart 5 software (AD Instruments, Lasec CPT, SA) through a BP transducer linking the arterial cannula to a PowerLab® via a BP amplifier. Randomized doses of drugs and extract were administered during the study.

### Dissolution and infusion of drugs

All drugs and plant extract were dissolved in 0.9% NS and administered per rat body weight in a volume not higher than 0.5 ml per dose. DMSO (2 to 3 drops) was added to the plant extracts for homogenous dilution and filtered before administration. Drugs were infused at a rate based on the body weight and dose over 3 minutes using an Ascor AP 22 syringe pump. The results were recorded within 3 minutes and drugs flushed with 0.5 ml normal saline. The BP was allowed to stabilize for 10 to 15 minutes, before further doses were infused. Six rats were used for each set of experiments.

### Experimental protocol

Time zero (0) to twenty (20) minutes, the SHR was collected from the animal room and anaesthetized. While waiting for the anesthesia to take effect, all equipments were checked to see if they are properly set up. Catheters were heparinised. Tracheal tube was inserted into the trachea and the cannulation of the jugular vein and femoral artery done. Subsequently, the recording of the experimental parameters began.

Time twenty one (21) to forty (40) minutes, the animal was allowed to recover from the surgical procedure. Drugs and/or extract to be administered were diluted.

Time forty one (41) minutes, the animal was stable and the first dose infused.

Time forty five (45) to sixty (60) minutes, flushing was done, followed by recovery of the animal from drug effect, until initial base line was reached. Subsequent doses were then infused using the same procedure as that of the first dose.

### Experimental conditions of the animals

To ascertain that the haemodynamic, respiratory and metabolic conditions of the rats were stable during the course of experiments, initial recordings of the cardiovascular parameters (SBP, DBP, MAP and HR) to be assessed during the study were done in a separate group of control SHRs over 3 hours, time much longer than the one needed for each experiment. During that period, no drug or plant extract was administered to the animal. Furthermore, in randomly selected rats, arterial blood sample (10 μl) was taken at the beginning (just after surgical preparation of the animal), and at the end of each experiment. Samples obtained were immediately sent in ice pack to PathCare Laboratory (Vet laboratory, Bellville, SA) for arterial blood gaz tests.

### Statistical analysis

The results obtained are presented as mean values (± SEM). Statistical significance between means was calculated using the Student’s t-test and *p* value <0.05 was considered significant.

### Ethics considerations

The study was approved by the ethics committee of the UWC (Ethics approval reference number 09/9/5) and was conducted according to the UWC rules and regulations in terms of animal experiments; and the European Community guidelines (EEC Directive of 1986; 86/609/EEC).

## Results

### Experimental conditions

No statistically significant difference was observed in the SBP, DBP, MAP, HR, respiratory rate and arterial blood gaz test results during 3 hours the experiments were conducted.

### Effect of A.gangetica on BP and HR in SHR

**Figure 1:**
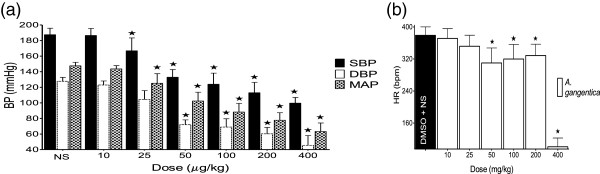
**Effect of *A. gangentica *on BP (a) and HR (b).** Values are presented as mean ±. SEM. * indicates statistical significance.

*A. gangetica* (10–400 mg/kg) significantly (<0.01), and dose-dependently decreased the maximum BP values obtained when compared to their respective values at baseline. *A. gangetica* also produced significant (<0.05) reductions in HR which were not dose-dependent (Figure 1).

### Effect of angiotensin I on the BP and HR in SHR

**Figure 2:**
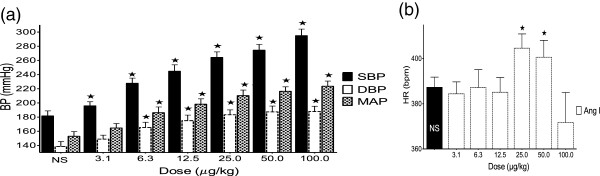
**Effect of angiotensin I on BP (a) and HR (b).** Values are presented as mean ±. SEM. * indicates statistical significance.

ANG I (3.1–100 μg/kg) significantly (<0.01), and dose-dependently increased the maximum BP values obtained when compared to their respective values at baseline. ANG I only produced significant (<0.05) change in HR at the 3^rd^ and 2^nd^ highest doses (25 and 50 μg/kg respectively, Figure 2).

### Effect of angiotensin I co-infused with A.gangetica on BP and HR in SHR

**Figure 3:**
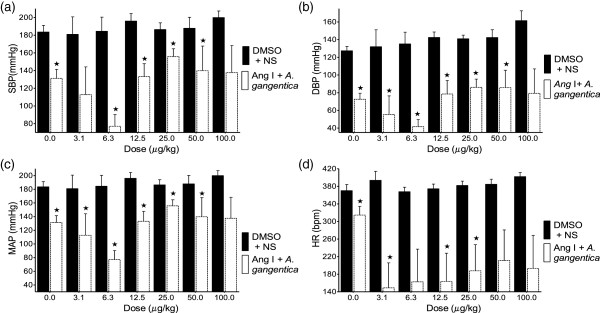
**Effect of ANG I co-infused with *A. gangetica *on the SBP (a), DBP (b), MAP (c), and HR (d).** Values are presented as mean ± SEM. * indicates statistical significance.

The effects of ANG I (3.1 - 100 μg/kg) on the SBP, DBP, and MAP was significantly (p<0.001) inhibited by co-infusion with *A. gangentica* (200 mg/kg). The effect of ANG I on the HR was also significantly inhibited by *A.gangetica* (Figure 3).

### Effect of angiotensin II on BP and HR in SHR

**Figure 4:**
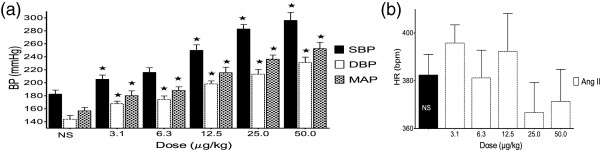
**Effect of angiotensin II on BP (a) and HR (b).** Values are presented as mean ± SEM. * indicates statistical significance.

ANG II (3.1 – 50.0 μg/kg) significantly increased the SBP, DBP, and MAP in a dose-dependent fashion (Figure 4).

### Effect of angiotensin II co-administered with A.gangetica on the BP and HR in SHR

**Figure 5:**
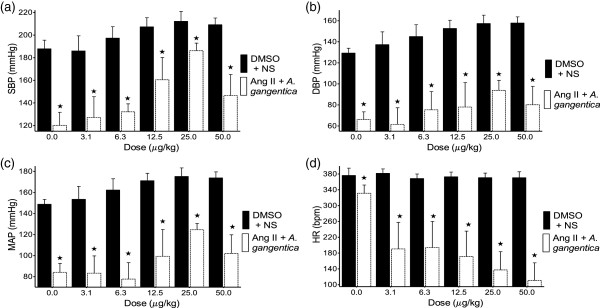
**Effect of ANG II co-infused with *A. gangetica *on the SBP (a), DBP (b), MAP (c), and HR (d).** Values are presented as mean ± SEM. * indicates statistical significance.

As shown in Figure 5, co-administration of *A. gangetica* (200 mg/kg) and ANG II (3.1 – 50.0 μg/kg), significantly (p<0.01) decreased the hypertensive effect of ANG II on the SBP, MAP and DBP, as well as its tachycardic effect.

## Discussion

### Effect of Asystasia gangetica on the blood pressure and heart rate

The results obtained in this study demonstrate that the ALE of *A. gangetica* decreases the BP in a dose dependent manner in SHR. The hypotensive effect observed (Figure 1) is concordant with the previous findings of Ramesar *et al.*[12] that both aqueous and methanol extracts of *A. gangetica* exhibited ACE inhibitory activity of 20% and 51% respectively *in-vitro*.

### Effect of angiotensin I on the blood pressure and heart rate

The increase in BP is due to the direct vasoconstrictory actions of ANG II produced from ANG I in the presence of ACE *in-vivo*[20,21]. Meanwhile, the increase in HR is due to significant potentiation of the sympathetic activity, as well as a direct excitatory action in the heart produced by high levels of ANG II [18,22-27]. The decrease in the effect of ANG I on the HR at the dose ≥ 100 μg/kg may simply be down to Ang II shifting the operating ‘set-point’ for the regulation of sympathetic outflow to a higher BP [24,28].

### Is the hypotensive effect of A.gangetica mediated by the inhibition of ACE?

The significant reductions in SBP, DBP and MAP produced by co-infusing the ALE of *A. gangetica* with ANG I (Figure 3) when compared to the BP values observed with infusing ANG I alone (Figure 2) can be attributed to *A. gangetica* inhibiting the conversion of ANG I into ANG II, a powerful vasoconstrictor [29-32]. The significant reductions in HR observed with the co-infusion of *A. gangetica* with increasing doses of ANG I (Figure 3), as opposed to the absence of change, or even significant increases observed at some doses with the infusion of ANG I alone (Figure 2), could be attributed to lesser quantities of ANG II being produced. Since the dose *of A. gangetica* is fixed, it is expected that less ANG II can be produced at the lower doses of ANG I, while more ANG II can be formed at the higher doses.

### Are angiotensin II receptors involved in the mechanism of action of Asystasia gangetica?

ANG II significantly increased the BP (Figure 4), due to the activation of ANG II receptors (AT _IA_) in the vascular smooth muscle cells, resulting in increased vasoconstriction, decreased renal blood flow and renal tubular sodium re-uptake, increased aldosterone and vasopressin secretion [30-36]. The co-administration of the ALE of *A. gangetica* with ANG II (Figure 5) significantly inhibited the effect of ANG II alone (Figure 4) on BP. This antihypertensive effect was associated with significant reductions in HR. This gives more credence to the opinion that *A. gangetica* contain chemical compounds, which act by blocking the actions of ANG II at its various receptors mentioned above.

#### Limitations of the study

The angiotensin II (AT_1_) receptor mediates all of the known physiological actions of ANG II in the cardiovascular, renal, neuronal, endocrine, hepatic and other target cells [29,33,37-39]. Therefore, it is important to investigate the specific action (s) of the crude ALE, and its constituents at each target cell, as well as, the involvement of the alpha-_1_, beta-_1_, and presynaptic alpha-_2_ adrenoceptors; cholinergic receptors and calcium channels in the mechanism of action of *A. gangetica*.

## Conclusions

*A. gangetica* decreases the blood pressure and heart rate in SHR. This action might be secondary to inhibition of ACE and the ANG II receptors. It may also involve a direct inhibitory action on the heart muscle.

## Abbreviations

A. gangetica: *Asystasia gangentica*; ACE: Angiotensin I converting enzyme; ALE: Aqueous leaf extract; ANG I: Angiotensin I; ANG II: Angiotensin II; AT1: Angiotensin II type 1 receptor; BP: Blood pressure; Bpm: Beats per minute; DBP: Diastolic blood pressure; DMSO: Dimethylsulfoxide; HR: Heart rate; MAP: Mean arterial pressure; mmHg: Millimetres of mercury; SBP: Systolic blood pressure; SEM: Standard error of mean; SHR: Spontaneously hypertensive rats; UWC: University of the Western Cape.

## Competing interests

The authors declare that they have no competing interests.

## Authors’ contributions

PM conceived the idea of the study, designed the study and participated in the acquisition, analysis, and interpretation of data. IR participated in the conceptualization of the design, acquisition, analysis, and interpretation of data, and carried out the technical aspect of the study. All authors read and approved the final manuscript.

## Authors’ information

PM has a PhD in Pharmacology, MMed in Cardiology, MBChB in medicine and B.Sc in human biology. PM is currently Professor of Pharmacology in the School of Pharmacy, University of the Western Cape, South Africa.

IR has a PhD in Pharmaceutical Sciences, M.Sc and B.Sc Honours degrees in Human Physiology. IR is currently a Senior Lecturer in Physiology, at the National University of Science and Technology, Bulawayo, Zimbabwe.

## Pre-publication history

The pre-publication history for this paper can be accessed here:

http://www.biomedcentral.com/1472-6882/13/283/prepub
